# Radiologist- and Surgeon-Performed Ultrasound (RSUS) Facilitates Minimally İnvasive Parathyroidectomy (MIP): Optimal Biochemical Parameters and Patient Outcomes

**DOI:** 10.3390/jcm14072279

**Published:** 2025-03-27

**Authors:** Vahit Mutlu, Mahmut Arif Yuksek, Zafer Pekkolay, Zeynep Yegin, Ibrahim Halil Yildirim, Omer Uslukaya

**Affiliations:** 1 Department of General Surgery, Faculty of Medicine, Uskudar University, 34662 Istanbul, Turkey; dr.vahitmutlu@gmail.com (V.M.); druslukaya@gmail.com (O.U.); 2 Department of General Surgery, Faculty of Medicine, Hitit University, 19040 Corum, Turkey; mahmutarifyuksek@gmail.com; 3 Department of Endocrinology, Faculty of Medicine, Dicle University, 21280 Diyarbakır, Turkey; drpekkolay@gmail.com; 4 Medical Laboratory Techniques Program, Vocational School of Health Services, Sinop University, 57000 Sinop, Turkey; 5 Department of Genetics, Faculty of Veterinary, Dicle University, 21280 Diyarbakir, Turkey; ihyildirim@gmail.com

**Keywords:** parathyroid adenoma, ultrasound, minimally invasive parathyroidectomy, hypercalcemia, parathormone

## Abstract

**Background/Objectives:** The high success rate of minimally invasive parathyroidectomy (MIP) is dependent upon the correct preoperative localization of the solitary parathyroid adenoma (SPA). Various studies have focused on comparisons of radiologist-performed ultrasound (RUS) and surgeon-performed ultrasound (SUS) to increase the sensitivity rate of US. However, the efficiency of radiologist- and surgeon-performed ultrasound (RSUS) before MIP has not frequently been reported. We aimed to evaluate the efficiency of RSUS in clinical practice. **Methods:** In total, 122 patients (107 females, 15 males, mean age: 47.62 ± 15.75 years) with SPA were enrolled in our study design. The patients underwent preoperative ultrasonography (US) and technetium-99-sestamibi scintigraphy. Patient data including demographic characteristics, levels of biochemical parameters (parathyroid hormone (PTH), total serum calcium and phosphorus levels), operation time, and length of hospital stay were recorded. **Results:** MIP was performed with success under local anesthesia following the accurate localization of the adenomas by RSUS. The mean operation time was 20.00 ± 3.87 min. The mean preoperative serum PTH, calcium, and phosphorus levels were 525.69 ± 1050.92 pg/mL, 11.38 ± 1.22 mg/dL, and 2.53 ± 0.60 mg/dL, respectively. The decline in the perioperative PTH and calcium levels reflecting a cure was observed on the first postoperative day. Postoperative sixth month evaluations of the PTH and calcium levels confirmed the significant decrease, reflecting the therapeutic cure. Since no complications occurred, the hospital discharge process was carried out on the same day. **Conclusions:** RSUS is a beneficial adjunctive tool to facilitate MIP, and it achieved satisfactory therapeutic success in all the patients.

## 1. Introduction

As a common endocrine disorder, primary hyperparathyroidism (PHPT) stems from the autonomous production of the parathyroid hormone (PTH) and is characterized by several clinical and biochemical manifestations [1]. The hypersecretion of PTH from enlarged parathyroid gland(s) results in skeletal, kidney, and gastrointestinal problems. PTH is a major hormone responsible for calcium homeostasis. Therefore, its hypersecretion results in hypercalcemia, which can even be life-threatening in some conditions. PHPT occurs in nearly 1% of the population, and it shows a tendency to rise in individuals who are over 60 years old [2,3,4,5]. Many more women than men (~4:1 ratio) develop this disease, especially in the early postmenopausal years, dependent upon estrogen loss [6].

PHPT is one of the most frequent endocrine disorders after diabetes and thyroid gland diseases [7]. In addition to a wide range of metabolic changes, it was reported that patients with PHPT had neurocognitive dysfunctions compared to the general population [8,9]. Nevertheless, a successful parathyroidectomy can significantly increase the quality of life, even in a month [10]. In a life quality evaluation study of parathyroidectomy, improvements in physical and mental health components were reported at 50% and 76%, respectively [11]. Parathyroidectomy was reported to significantly improve bone mineral density (BMD) and remodeling biomarkers in women with osteopenia [12]. A recent systematic review and meta-analysis study reported the lessened risk of both total and cardiovascular (CV) death following parathyroidectomy [13]. The systemic effects of PHPT may be related to inflammatory cytokines. A recent study reported that serum IL-6, C-reactive protein, and monocyte chemoattractant protein-1 (MCP-1) were higher in patients with PHPT, and parathyroidectomy decreased the serum IL-6 and MCP-1 levels [14].

The traditional curative approach is parathyroidectomy by collar incision which allows four-gland (typically) exploration with high success. However, a very high proportion of PHPT cases are related to an isolated parathyroid adenoma. In fact, a significant proportion of cases affected by PHPT (80–90%) are solely solitary parathyroid adenoma (SPA). The remaining groups consist of multiglandular disease (2–5%), chief cell hyperplasia (15–20%), and extremely rare cases of parathyroid carcinoma (<1%) [3,15,16]. Approximately 10% of PHPT cases have a genetic background including mutations in several genes. The key genes are *MEN1*, *CDC73*, and *RET*, which are also associated with other neoplasms. Young-onset PHPT, multiglandular PHPT, personal history of other features associated with PHPT genes, family history of PHPT and/or related tumors, and PT carcinoma indicate the necessity for genetic screening [5,17]. In addition, the investigation of potential candidate genes for primary hyperparathyroidism is of utmost importance. For instance, a meta-analysis study revealed that variants of the calcium-sensing receptor (CASR) gene (rs1081725 and rs1042636) may elevate the PHPT risk and thus have the potential to be used as molecular markers for early diagnosis [18]. A very recent study highlighted that PHPT is characterized by a unique microRNA (miRNA) signature. It was found that, of the investigated miRNAs, nine miRNAs (miR-335-5p, miR-130b-3p, miR-125b-5p, miR-23a-3p, miR-152-3p, miR-582-5p, miR-144-5p, miR-320a, and miR-19b-3p) were differentially expressed in PHPT compared to matched controls. In addition, all these differentially expressed miRNAs were significantly associated with levels of serum PTH, and eight of the nine correlated with calcium levels [19].

Diagnosis of PHPT is carried out through laboratory evaluations. The calcium and associated PTH levels are too high, while the phosphate levels are low. A 24 h urinary collection confirms the diagnosis by excluding familial hypocalciuric hypercalcemia [4]. Calcium homeostasis is carefully regulated by three calcium-regulating hormones, namely PTH, 1,25-dihydroxy vitamin D, and calcitonin. A decrease in serum calcium is sensed by the CASR in the chief cells of the parathyroid glands, which increases PTH synthesis and secretion. PTH increases can finally result in enhanced intestinal absorption of calcium and phosphate as well as mobilization of calcium from the skeleton [20]. A high postoperative serum PTH may predict persistent disease and necessitate long-term surveillance for recurrence [21,22], also reflecting the ineffectiveness of surgical treatment.

The symptoms of PHPT are widely variable. Nearly half or more patients with PHPT are asymptomatic. Specific symptoms can be kidney stones or bone diseases. Urinary, musculoskeletal, gastrointestinal, cardiovascular, neuromuscular, and neuropsychiatric symptoms may be observed in changing frequencies. Reduced bone mineral density and nephrolithiasis are the major clinical pathological conditions. Luckily, the detection of asymptomatic cases has increased, and thus the incidence of renal complications can be better managed [5,7,23]. To better predict the clinical outcomes of PHPT, evaluation of serum magnesium (Mg) may also be helpful. A recent study reported the possible effects of hypomagnesemia in patients with PHPT; the patients with hypomagnesemia had higher rates of symptomatic disease, kidney stones, and osteoporosis with a decrease in serum vitamin D levels [24].

Since more than 80% of cases with PHPT are related to a single solitary adenoma, the cure can be achieved with the excision of the affected gland. Therefore, the traditional surgical treatment of PHPT has shifted from bilateral neck exploration (BNE) to a more focused approach called minimally invasive parathyroidectomy (MIP). MIP and BNE have comparable cure rates in PHPT. However, in a suitable patient group, MIP has more advantages than BNE. These advantages are a shorter duration of the operation, a decreased post-operative hypocalcemia rate, a shorter hospital stay, decreased morbidity, decreased cost, and improved cosmesis [25,26,27].

The high success rate of MIP is dependent upon the accurate preoperative localization of the adenoma. The most commonly used imaging methods comprise a parathyroid scan with technetium Tc 99m MIBI (sestamibi scan) and cervical ultrasonography (US). Before operative intervention in PHPT, the use of both is a common approach. Less common imaging techniques include computed tomography (CT) and magnetic resonance imaging (MRI) [27,28]. US offers several advantages such as the avoidance of ionizing radiation, a portable study design, and a low cost. On the other hand, it can achieve its highest accuracy in the hands of surgeons very familiar with anatomical expertise [29]. In order to increase the sensitivity of US, several studies have focused on comparisons of radiologist-performed ultrasound (RUS) and surgeon-performed ultrasound (SUS). The proven efficiency of radiologist- and surgeon-performed ultrasound (RSUS) before MIP was evaluated a decade ago with a restricted number of patients [30]. However, it seems that RSUS is not a regular approach in clinics, and more studies are needed to reach to a consensus.

The aim of the study is to evaluate the results and feasibility of radiologist- and surgeon-performed ultrasound (RSUS) before MIP in a relatively high number of patients (122 patients with solitary parathyroid adenoma (SPA)-related PHPT). Preoperative and postoperative biochemical values (PTH, total serum calcium and phosphorus levels) of the cases were recorded, and a surgical cure was defined in terms of biochemical parameters, achieving normocalcemia 6 months postoperatively.

## 2. Materials and Methods

### 2.1. Participants

**Figure 1:**
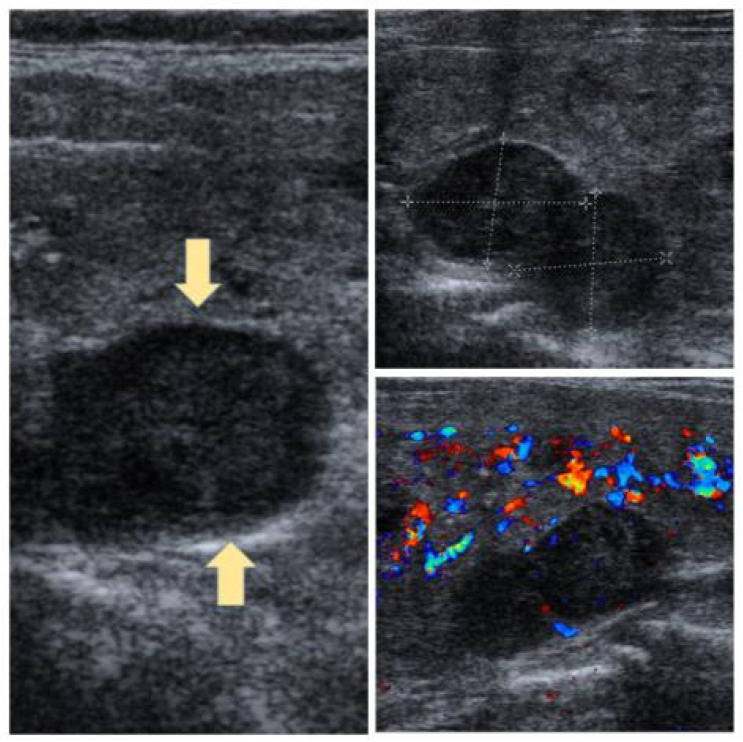
Ultrasound imaging of parathyroid adenoma.

From 2019 to 2024, 265 consecutive patients were operated on for PHPT at the General Surgery Clinics. PHPT was caused by SPA in 122 patients, and these patients were enrolled in the study. The demographic characteristics of the patients, surgery duration, and hospital stay were recorded retrospectively from the hospital database. All patients were confirmed to have SPA-related PHPT by biochemical tests and imaging methods (Figure 1). MIP with local anesthesia and intravenous sedation was preferred in this patient group. The exclusion criteria were multiple gland disease, multiple endocrine neoplasia syndrome or suspected parathyroid carcinoma, patients allergic to local anesthetic agents, uncooperative patients requiring general anesthesia, and thyroid disease requiring preoperative intrathyroidal parathyroid or thyroidectomy. Intraoperative parathyroid hormone measurement was not used. The size and weight of all the excised glands were measured. The removed specimens were fixed in 10% formalin solution and sent to pathology. The frozen section was not used, and the operation was terminated with excision of the gland.

### 2.2. Biochemical Evaluations

The parathyroid hormone was studied using the electrochemiluminescence method with the Roche Cobas E-601 (Basel, Switzerland) device. Calcium and phosphate levels were studied using the photometric method with the Beckman AU-5800 (Pasadena, CA, USA) device. The biochemical parameters of the patients were recorded. The parathyroid hormone (PTH) and total serum calcium and phosphorus levels of all cases were analyzed before surgery. These biochemical parameters were evaluated regularly on the first postoperative day, the 2nd week, 3rd month, and 6th month.

### 2.3. Localization Study

**Figure 2:**
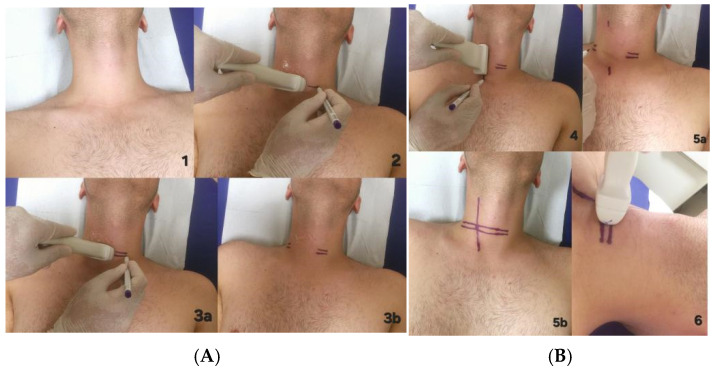
(**A**,**B**) planning of surgical procedure with ultrasonography.

Ultrasonography (US) was the diagnostic procedure used. US was performed one day before the surgery in the Radiology department with the cooperation of the surgeon and radiologist. Since ultrasound is a person-dependent method, working with a radiologist familiar with head and neck imaging increases the sensitivity, and we acted accordingly. The patient was processed in hyperextension as during the operation (Figure 2A(1)). The adenoma was visualized with the ultrasound linear probe (Figure 2A(2)). The upper border of the lesion in the transverse plane was marked with the probe. Then, the lower border was marked (Figure 2A(3a,3b)). The long axis of the lesion was determined by US (Figure 2B(4)). By combining all the points, the skin projection of the lesion was determined (Figure 2B(5a,5b)). In all procedures, the US probe should be perpendicular to the neck axis (Figure 2B(6)). Finally, the process was completed by determining the distance of the adenoma from the skin.

### 2.4. Anesthesia Technique

The patients were monitored in the preoperative preparation room before surgery. Patients were sedated by administering 0.02–0.05 mg/kg midazolam intravenously. Later, the patients were transferred to the operating room with a monitor. After standard anesthesia monitoring, patients were given 1 µg/kg fentanyl and 0.5 mg/kg propofol intravenously (IV). After an adequate level of sedoanalgesia was provided, local anesthetics were applied for infiltration anesthesia. For infiltration anesthesia, 1 mg/kg of 0.25% bupivacaine and 1 mg/kg of 1% lidocaine were prepared, and half the dose was applied to the incision area. The remaining half was applied to the adjacent tissues and deeper levels. When general anesthesia was necessitated for any reason, the patients were intubated by the anesthesiologists.

### 2.5. Surgery Technique

With a support placed vertically under the scapulas of the patient lying on their back, the head was lowered backwards, and the neck was brought into a hyperextension position. In the hyperextension position of the patient’s head, the back of the neck was filled with support structures. In order to provide a better field of view, his/her head was raised 20–40 degrees by positioning the operating room table. The skin incision was made at the location previously marked under ultrasound guidance between the suprasternal notch and the thyroid cartilage. The incision was preferably made parallel to the natural lines of the patient’s neck and with the ends slightly sloping upwards. The length of the skin incision was usually between 1.5 and 2 cm but could vary depending on the size of the adenoma and the patient’s body structure. After passing the skin, subcutaneous tissue, and platysma layers, the subplatysmal flap was raised from the avascular area between the platysma and the neck median fascia. Flap dissection was continued in the superior, inferior and both lateral directions for approximately 2 cm. After passing the platysma muscle, the muscles that emerge and the fascia covering these muscles were passed with blunt and sharp dissection in the direction of the angle where the adenoma was detected by the preoperative ultrasound probe, and the adenoma was reached. The adenoma was removed by freeing it from the surrounding tissues with sharp and blunt dissection. The time from the beginning of the incision to the suturing of the skin was defined as the operative time.

### 2.6. Statistical Analyses

Statistical analyses were performed using SPSS software program version 22.0 for Windows. Categorical variables were expressed as numbers (percentages) and continuous variables as means ± standard deviation. The paired sample t-test or Wilcoxon rank test was used to define differences between the preoperative and postoperative PTH, Ca, and P after determining normality with the Kolmogorov–Smirnov test. A *p* value < 0.05 was considered as statistically significant.

## 3. Results

In total, 107 of 122 patients (87.7%) were women. The average age was 47.27 ± 15.62 (range 17–88) for women, 50.13 ± 16.96 (range 17–80) for men, and 47.62 ± 15.75 in total. The distribution of the excised adenomas was 57.4% (n = 70) on the right side and 42.6% (n = 52) on the left side. All patients underwent intervention with local anesthesia. No patient required conversion to general anesthesia. The patient demographic data and clinical details are shown in Table 1.

All patients had biochemically confirmed PHPT and longitudinally localized single gland disease supported by sestamibi scan (SS) and US. US was performed on all patients. While parathyroid adenoma was detected by US in all patients, SS could not identify parathyroid adenoma in nine patients (7.4%). According to the US and operative findings, the localization of parathyroid adenomas was 63 (51.6%) in the lower right region, 7 (5.7%) in the upper right region, 43 (35.2%) in the lower left region, and 9 (7.4%) in the left. All adenomas detected by SS were compatible with US and operation localization.

The average weight of the excised parathyroid adenomas was 2.06 ± 2.69 g (range 0.2–17.6 g). The average largest diameter was 1.99 ± 0.91 cm (range 0.6–7 cm). Preoperative and postoperative serum calcium, phosphorus, and PTH values are represented in Table 2. The ranges of biochemical parameters are also represented in Table 3. While postoperative Ca and PTH levels were significantly lower than preoperative values (*p* < 0.001), the phosphorus value was significantly higher (*p* = 0.015). The elevated preoperative serum PTH and calcium levels returned to normal by the first postoperative visit. All patients were followed for at least 6 months. The surgical cure was defined in terms of biochemical parameters as achieving normocalcemia 6 months postoperatively. Therapeutic success was provided in all patients according to the biochemical results (serum PTH, calcium, and phosphorus). This demonstrated that the method applied was undisputedly successful.

The mean surgery time was 20.00 ± 3.87 min (range 15–32 min), and no patient experienced any complications. Following the operation, the patients were either discharged from the hospital or transferred to the Endocrinology Department.

## 4. Discussion

PHPT is a disorder in which one or more of the parathyroid gland/glands are overactivated. The most frequent type encountered is the parathyroid adenoma of a single gland, which can now be managed much better with a revolutionized approach called minimally invasive procedure (MIP). MIP is very advantageous in a suitable patient group, since it shortens the operative and anesthesia time and prevents unnecessary mobilization of neck structures, thus lowering the rate of recurrent laryngeal nerve damage. Other advantages include a shortened hospitalization time, lower costs, less complications, low morbidity, and a better cosmetic result [31,32,33]. The success rate of MIP is closely dependent upon the use of appropriate imaging techniques for accurate preoperative localization. Primary localization techniques are technetium-99m sestamibi scanning (MIBI) and ultrasound (US) of the neck [34]. The sensitivities of the neck US and MIBI are in the ranges of 38–96% and 56–95%, respectively. However, the combination of MIBI and neck US results in an increase in the sensitivity (74–90%) for localizing a parathyroid adenoma [35]. While the use of technological adjuncts varies among experienced surgeons, the results of a survey of Asia–Pacific parathyroid surgeons showed that 75% of surgeons prefer dual imaging with sestamibi and ultrasound. Ten percent of surgeons use parathyroid four-dimensional computed tomography (4DCT) as first-line imaging, more commonly in East Asia, since its algorithm can define negative or contradictory sestamibi and ultrasound results [36]. US is safe since it eliminates the use of any contrast material or dye and noninvasive. US has a high diagnostic sensitivity of 89% and a positive predictive value (PPV) of 97%, making it a highly efficient first-line imaging technique before MIP. The ability of the sestamibi scan to predict the precise site of the adenoma was 90%, while it reached 95% with sonography [37]. It was reported that US correctly diagnosed 45% of patients that technetium-99m sestamibi scanning (MIBI) missed [34]. However, operator-dependence may be a limitation for this valuable imaging method [31,38]. To overcome this limitation, several studies have focused on comparisons of radiologist-performed ultrasound (RUS) and surgeon-performed ultrasound (SUS). Centers with RUS and MIBI scanning, as well as the use of SUS, facilitated accurate preoperative localization of solitary parathyroid adenomas [39]. It was reported that SUS was able to localize 94% of adenomas in sestamibi scan-negative patients [40]. Preoperative SUS has the benefit of characterizing concurrent thyroid pathology, a lower cost, a lack of ionizing radiation, and a high specificity. Moreover, it is non-invasive and can be conveniently performed in both the surgeon’s rooms and in the operating theater. The anatomical expertise of the surgeon could correlate to greater accuracy in terms of localization. If SUS could not identify a solitary adenoma, 4DCT could also be preferred to sestamibi as the second-line imaging modality [29,39,41]. SUS had a sensitivity of 79.4% and a PPV of 97.0%, MIBI had a sensitivity of 60.0% and a PPV of 95.3%, and RUS had a sensitivity of 65.5% and a PPV of 98.7%. When used as a second-line modality, 4DCT had a sensitivity of 76.9% and a PPV of 100%. Both SUS and 4DCT were found to be more sensitive than MIBI to correctly localize parathyroid lesions. However, radiation is an important issue with 4DCT, and it is particularly efficient at the detection of the smaller lesions found in multiglandular disease [39]. In institutions with dedicated parathyroid and thyroid radiologists, the accuracy of RUS is as high as SUS. Overall, RUS was reported to correctly localize the parathyroid adenomas in 82% of patients while SUS correctly localized the abnormal adenomas in 83% of cases. The high sensitivity of SUS as reported in some studies could be associated with the surgeon’s familiarity with neck anatomy. Therefore, it may be helpful for units without specialized radiologists. It is also a beneficial adjunctive tool to sestamibi localization to facilitate MIP. Moreover, concomitant thyroid pathology can also be investigated [35]. A recent study highlighted the changing sensitivities of RUS and SUS. Sestamibi, RUS, and SUS were reported to have sensitivities of 59.4%, 43.75%, and 73.8%, respectively. SUS had a statistically significant increased sensitivity compared with RUS [42]. A systematic review study consisting of a total of 5282 patients with a mean follow-up of 33.5 months reported the overall mean recurrence and cure rates with MIP as 1.6 (range 0–3.5) and 96.9 (95.5–100)%, respectively [43]. In the literature, with an innovative approach, Uslukaya et al. [30] successfully performed radiologist- and surgeon-performed ultrasound (RSUS) before MIP. Though the number of patients in the study group was relatively small (30 patients with SPA), very promising results were obtained. Adenomas were successfully localized by US in all patients, and MIP was successfully performed under local anesthesia. The mean operation time was shorter, and the postoperative PTH and calcium values were significantly decreased implying a biochemical cure. All patients were discharged from the hospital in the same day without any complications.

Our study design consisted of 122 patients with biochemically confirmed PHPT and longitudinally localized single gland disease supported by sestamibi and US. US was performed one day before the surgery in the Radiology Department with the cooperation of the surgeon and radiologist (radiologist- and surgeon-performed ultrasound (RSUS)). The cooperative imaging technique is one of the most powerful aspects of the study’s design. Therapeutic cure was achieved in the patients based on preoperative–postoperative biochemical parameter comparisons (Table 2 and Table 3). Successful implementation of RSUS to facilitate accurate localization and then MIP reached maximum satisfactory outcomes in the patients. With this method, since there is no need for the equipment and personnel required for video assistance and wire marking, the operation time is shortened, and the cost is reduced. Additionally, since these materials cannot be found in every clinic, their applicability is limited. In addition to the therapeutic success proven by the biochemical parameters, the operation time was quite short, no patient required conversion to general anesthesia, and no patient faced any complications such as recurrent laryngeal nerve damage, hematoma, or injury to nearby organs. We performed a defined surgery, minimally invasive parathyroidectomy. The difference is that the radiologist and the surgeon performed the ultrasound together, as a method to facilitate the surgery. Therefore, there was no need for a control group that would require a different diagnosis and approach, and this does not constitute a limiting factor in the study.

The high success rate achieved with minimally invasive parathyroidectomy (MIP) depends on the correct preoperative localization of the solitary parathyroid adenoma (SPA). We achieved significant results in the detection and treatment of pathology with ultrasonography performed by the surgeon and the radiologist. The basis of treatment for primary hyperparathyroidism is to find the adenoma and normalize the calcium and phosphorus levels. We demonstrated this situation effectively with this method. It is also worth remembering that ultrasound technology is open to further development with new technologies such as deep learning (DL)-based methods, as highlighted in some recent studies [44,45].

As a result, ultrasound, when performed jointly by the radiologist and surgeon, allows the surgeon to directly image the parathyroid adenoma with the patient’s position in surgery and correlate this with other important anatomical markers in the thyroid and neck by planning in real time for a multitude of possibilities during or before surgery. Additionally, it increases the reliability by determining the starting point on the neck for surgery. Therefore, when comparing the ultrasound performed by the surgeon alone or the ultrasound performed by the radiologist alone, the ultrasound performed by the two in combination (RSUS) significantly shortens the operation time.

## 5. Conclusions

Minimally invasive parathyroidectomy (MIP) is a valuable method of choice in well-selected patients. Our data suggest RSUS would be very accurate and helpful in operative planning. The minimally invasive procedure was very efficient based on the postoperative biochemical parameters compared to the elevated preoperative biochemical values. Following RSUS, MIP can be safely and efficiently performed in routine use without any apparent complications.

## Figures and Tables

**Table 1:** Patient characteristics and details.

Total number	122
Age (years ± SD)	47.62 ± 15.75
Female, n (%)	107 (87.7%)
Preoperative biochemistry	
Calcium (normal 8.4–10.2 mg/dL)	11.38 ± 1.22
Phosphorus (normal 3.2–5.5 mg/dL)	2.53 ± 0.60
Parathyroid hormone (normal 15–65 pg/mL)	525.69 ± 1050.92
Etiology, n (%)	
Single adenoma	122 (100%)
Surgical procedure	
MIP	122
Operation time	
Minimum	15.00
Maximum	32.00
Mean ± SD	20.0082 ± 3.87511
Mean gland weight, g	2.06 ± 2.69
Mean maximal diameter (cm)	1.99 ± 0.91

MIP: minimally invasive parathyroidectomy; SD: standard deviation.

**Table 2:** Assessments of biochemical parameters in patients with SPA.

Parameter	Preoperative	Postoperative First Day	Postoperative Sixth Month	Normal Range
PTH	525.69 ± 1050.92	46.81 ± 97.54	58.31 ± 50.85	15–65 pg/mL
Calcium	11.38 ± 1.22	8.97 ± 1.01	8.98 ± 0.78	8.4–10.2 mg/dL
Phosphorus	2.53 ± 0.60	3.93 ± 5.72	3.61 ± 0.71	3.2–5.5 mg/dL

PTH: parathyroid hormone.

**Table 3:** The ranges of the investigated biochemical parameters.

Parameter	Range
Preoperative Ca	8.6–18.5 mg/dL
Postoperative Ca (1st day)	6.9–12.3 mg/dL
Postoperative Ca (6th month)	6.3–10.2 mg/dL
Preoperative PTH	77–4233 pg/mL
Postoperative PTH (1st day)	3.43–903 pg/mL
Postoperative PTH (6th month)	9.93–100 pg/mL
Preoperative phosphorus	1.2–5.2 mg/dL
Postoperative phosphorus (1st day)	1.2–5.2 mg/dL
Postoperative phosphorus (6th month)	1.7–5.5 mg/dL

## Data Availability

The original contributions presented in this study are included in the article. Further inquiries can be directed to the corresponding author.

## References

[1] Madkhali T., Alhefdhi A., Chen H., Elfenbein D. (2016). Primary hyperparathyroidism. Ulus. Cerrahi Derg..

[2] Haciyanli M., Genc H., Damburaci N., Oruk G., Tutuncuoglu P., Erdogan N. (2009). Minimally invasive focused parathyroidectomy without using intraoperative parathyroid hormone monitoring or gamma probe. J. Postgrad. Med..

[3] Schneider R., Hinrichs J., Meier B., Walz M.K., Alesina P.F. (2019). Minimally Invasive Parathyroidectomy without Intraoperative PTH Performed after Positive Ultrasonography as the only Diagnostic Method in Patients with Primary Hyperparathyroidism. World J. Surg..

[4] Van Den Heede K., Bonheure A., Brusselaers N., Van Slycke S. (2022). Long-term outcome of surgical techniques for sporadic primary hyperparathyroidism in a tertiary referral center in Belgium. Langenbecks Arch. Surg..

[5] Kochman M. (2023). Primary hyperparathyroidism: Clinical manifestations, diagnosis and evaluation according to the Fifth International Workshop guidelines. Reumatologia.

[6] Silva B.C., Cusano N.E., Bilezikian J.P. (2024). Primary hyperparathyroidism. Best Pract. Res. Clin. Endocrinol. Metab..

[7] Kowalski G.J., Buła G., Żądło D., Gawrychowska A., Gawrychowski J. (2020). Primary hyperparathyroidism. Endokrynol. Pol..

[8] Febrero B., Ruiz-Manzanera J.J., Ros-Madrid I., Teruel E., Rodríguez J.M. (2023). Quality of life, mood and sleep quality in patients with primary hyperparathyroidism. Impact of socio-personal and clinical profile. Ann. Endocrinol..

[9] Lightle W.R., Zheng F., Makris K.I., Grogan R., Suliburk J. (2024). Objectively measured cognitive dysfunction in patients with primary hyperparathyroidism improves after parathyroidectomy. Surgery.

[10] Christensen J.W., Thøgersen K.F., Jensen L.T., Krakauer M., Kristensen B., Bennedbæk F.N., Zerahn B. (2022). Changes in quality of life 6 months after parathyroidectomy for primary hyperparathyroidism. Endocr. Connect..

[11] Somuncu E., Kara Y. (2021). The effect of parathyroidectomy on quality of life in primary hyperparathyroidism: Evaluation with using sf-36 and phpqol questionnaire. Endocr. J..

[12] Frey S., Gérard M., Guillot P., Wargny M., Bach-Ngohou K., Bigot-Corbel E., Moreau N.R., Caillard C., Mirallié E., Cariou B. (2024). Parathyroidectomy Improves Bone Density in Women with Primary Hyperparathyroidism and Preoperative Osteopenia. J. Clin. Endocrinol. Metab..

[13] Kong S.K., Tsai M.C., Yeh C.L., Tsai Y.-C., Chien M.-N., Lee C.-C., Tsai W.-H. (2024). Association between primary hyperparathyroidism and cardiovascular outcomes: A systematic review and meta-analysis. Bone.

[14] Meng L., Shapses S.A., Wang X. (2024). Parathyroidectomy Reduces Inflammatory Cytokines and Increases Vitamin D Metabolites in Patients With Primary Hyperparathyroidism. Endocr. Pract..

[15] Butt H.Z., Husainy M.A., Bolia A., London N.J. (2015). Ultrasonography alone can reliably locate parathyroid tumours and facilitates minimally invasive parathyroidectomy. Ann. R. Coll. Surg. Engl..

[16] Chakrabarty N., Mahajan A., Basu S., D’Cruz A.K. (2024). Imaging Recommendations for Diagnosis and Management of Primary Parathyroid Pathologies: A Comprehensive Review. Cancers.

[17] De Sousa S.M.C., Carroll R.W., Henderson A., Burgess J., Clifton-Bligh R.J. (2022). A contemporary clinical approach to genetic testing for heritable hyperparathyroidism syndromes. Endocrine.

[18] Wang X.M., Wu Y.W., Li Z.J., Zhao X.H., Lv S.M., Wang X.H. (2016). Polymorphisms of CASR gene increase the risk of primary hyperparathyroidism. J. Endocrinol. Investig..

[19] Wachtel H., Ermer J.P., Fraker D.L., Kelz R.R., Kelly T.L.A., Hackl M., Levine M.A. (2024). Circulating MicroRNA as a Potential Biomarker for Skeletal Disease in Primary Hyperparathyroidism: A Case-control Study. Ann. Surg..

[20] Khan A.A. (2013). Medical management of primary hyperparathyroidism. J. Clin. Densitom..

[21] Dream S., Kim G.Y., Doffek K., Yen T.W., Carroll T., Shaker J., Evans D.B., Wang T.S. (2024). Persistent elevation of parathyroid hormone after curative parathyroidectomy: A risk factor for recurrent hyperparathyroidism. World J. Surg..

[22] Tamski J., Hakala T., Huhtala H., Metso S. (2024). Clinical characteristics and outcomes of patients operated for primary hyperparathyroidism at Tampere University Hospital in 2017–2018. Scand. J. Surg..

[23] Zanocco K.A., Yeh M.W. (2017). Primary Hyperparathyroidism: Effects on Bone Health. Endocrinol. Metab. Clin. N. Am..

[24] Düğer H., Uçan B., Çalışkan M., Bostan H., Demirci T., Gül Ü., Çakal E., Kızılgül M. (2024). Hypomagnesemia may be associated with symptomatic disease in patients with primary hyperparathyroidism. Endocrine.

[25] Udelsman R. (2002). Six hundred fifty-six consecutive explorations for primary hyperparathyroidism. Ann. Surg..

[26] Arora S., Balash P.R., Yoo J., Smith G.S., Prinz R.A. (2009). Benefits of surgeon-performed ultrasound for primary hyperparathyroidism. Langenbecks Arch. Surg..

[27] Reilly D.J., Chew G.L., Eckhaus J., Smoll N.R., Farrell S.G. (2016). Outcomes for minimally invasive parathyroidectomy: Widening inclusion criteria based on preoperative imaging results. ANZ J. Surg..

[28] Al-Kurd A., Levit B., Assaly M., Mizrahi I., Mazeh H., Mekel M. (2018). Preoperative localization modalities in primary hyperparathyroidism: Correlation with postoperative cure. Surgery.

[29] El Lakis M., McKee J., Mohan N. (2024). Surgeon Performed Ultrasound in Preoperative Parathyroid Localization: A Real-World Experience in a Community Hospital. J. Surg. Endocrinol..

[30] Uslukaya O., Gumus M., Tasdemir B., Goya C., Kilinc F., Oguz A., Turkoglu A., Bozdag Z. (2015). Improvement of minimally invasive parathyroidectomy outcomes by real time ultrasonography performed by a surgeon and radiologist team. Med. Ultrason..

[31] Gilat H., Cohen M., Feinmesser R., Benzion J., Shvero J., Segal K., Ulanovsky D., Shpitzer T. (2005). Minimally invasive procedure for resection of a parathyroid adenoma: The role of preoperative high-resolution ultrasonography. J. Clin. Ultrasound.

[32] Prasannan S., Davies G., Bochner M., Kollias J., Malycha P. (2007). Minimally invasive parathyroidectomy using surgeon-performed ultrasound and sestamibi. ANZ J. Surg..

[33] Aksoy S.Ö., Adiyaman S.C., Çevlik A.D., Durak M.G., Seçil M., Sevinç A.I. (2021). Intra-operative parathyroid hormone evaluation is superior to frozen section analysis in parathyroid surgery. Am. J. Otolaryngol..

[34] Adler J.T., Chen H., Schaefer S., Sippel R.S. (2011). What is the added benefit of cervical ultrasound to ⁹⁹mTc-sestamibi scanning in primary hyperparathyroidism?. Ann. Surg. Oncol..

[35] Soon P.S., Delbridge L.W., Sywak M.S., Barraclough B.M., Edhouse P., Sidhu S.B. (2008). Surgeon performed ultrasound facilitates minimally invasive parathyroidectomy by the focused lateral mini-incision approach. World J. Surg..

[36] Chen R., Oh H.B., Parameswaran R., Gorelik A., Miller J.A. (2019). Practice Patterns in Parathyroid Surgery: A Survey of Asia-Pacific Parathyroid Surgeons. World J. Surg..

[37] Ben Haim M., Zwas S.T., Munz Y., Rosin D., Shabtai E.L., Kuriansky J., Olchovsky D., Zmora O., Scarlat A., Ayalon A. (2003). Focused, minimally invasive radio-guided parathyroidectomy: A feasible and safe option for elderly patients with primary hyperparathyroidism. Isr. Med. Assoc. J..

[38] Erdemir R.U., Taşdöven İ., Bayraktaroğlu T., Çakmak G.K. (2021). Intraoperative ultrasound imaging and sono-scintigraphic concordance improves success rates of minimally invasive parathyroidectomy. Turk. J. Med. Sci..

[39] Binks M., Burrows D., Littlejohn D. (2019). A rural perspective on minimally invasive parathyroidectomy: Optimal preoperative imaging and patient outcomes. ANZ J. Surg..

[40] Livingston C.D., Victor B., Askew R., Abikhalid J., Meynig J., Lindsey M., Jones L. (2008). Surgeon-performed ultrasonography as an adjunct to minimally invasive radio-guided parathyroidectomy in 100 consecutive patients with primary hyperparathyroidism. Endocr. Pract..

[41] Eller M., Dave A., Johnson C., Fingeret A.L. (2021). Accuracy of 4-Dimensional Computed Tomography for Localization in Primary Hyperparathyroidism. J. Surg. Res..

[42] Habib A., Molena E., Snowden C., England J. (2023). Efficacy of surgeon-performed, intra-operative ultrasound scan for localisation of parathyroid adenomas in patients with primary hyperparathyroidism. J. Laryngol. Otol..

[43] Ishii H., Mihai R., Watkinson J.C., Kim D.S. (2018). Systematic review of cure and recurrence rates following minimally invasive parathyroidectomy. BJS Open.

[44] Luan S., Yu X., Lei S., Ma C., Wang X., Xue X., Ding Y., Ma T., Zhu B. (2023). Deep learning for fast super-resolution ultrasound microvessel imaging. Phys. Med. Biol..

[45] Yu X., Luan S., Lei S., Huang J., Liu Z., Xue X., Ma T., Ding Y., Zhu B. (2023). Deep learning for fast denoising filtering in ultrasound localization microscopy. Phys. Med. Biol..

